# CARDIAC SPECIFIC OVEREXPRESSION OF SERUM RESPONSE FACTOR ALTERS MITOCHONDRIAL STRUCTURE AND FUNCTION

**DOI:** 10.1093/geroni/igae098.3640

**Published:** 2024-12-31

**Authors:** Pankaj Patyal, Gohar Azhar, Ambika Verma, Xiaomin Zhang, Jeanne Y Wei

**Affiliations:** University of Arkansas for Medical Sciences, Little Rock, Arkansas, United States; University of Arkansas for Medical Sciences, Little Rock, Arkansas, United States; University of Arkansas for Medical Sciences, Little Rock, Arkansas, United States; University of Arkansas for Medical Sciences, Little Rock, Arkansas, United States; University of Arkansas for Medical Sciences, Little Rock, Arkansas, United States

## Abstract

Serum response factor (SRF) is a transcription factor required for the regulation of genes important for cardiac structure and function. Previously, we have shown that increased levels of SRF in transgenic mouse carrying human SRF under Myh6 promoter resulted in cardiomyopathy and early mortality. Although, there is a rapidly growing body of literature investigating the effects of aging on the heart and how age-related alterations affect cardiac and mitochondrial function, and underlying mechanisms remain unclear. Herein, we tested the hypothesis whether cardiac specific overexpression of SRF alters mitochondrial function. We utilized SRF transgenic mice at different postnatal ages to demonstrate temporal increase in the SRF expression in the heart. Western blot analysis demonstrate the significant increase in the BNP, marker for left ventricular cardiac hypertrophy (p < 0.01). Electron microscopy revealed mitochondrial abnormalities. Further, we used high-resolution respirometry to determine mitochondrial function in SRF overexpressed mouse heart. Our data indicate the significant decrease in the mitochondrial respiratory chain complexes (I-IV) respiratory rates and maximal electron transport system capacity (p < 0.01). The markers for mitochondria biogenesis PGC1α and PGC1β expression levels were reduced analyzed by western blot analysis (p < 0.05). Further, protein expression levels of Opa1 and DRP1, markers for mitochondrial fusion and fission were significantly reduced (p < 0.01). Our findings implicate that the increase in the SRF expression in cardiac aging induce mitochondrial alterations and functional decline. Thus, SRF is likely to be one of the downstream effectors of the signaling pathways involved in mediating mitochondrial function.

